# Comparison of knowledge, awareness, and behaviors toward oral cancer among dental students and dentists: an online cross-sectional questionnaire in Türkiye

**DOI:** 10.1186/s12903-024-04241-6

**Published:** 2024-04-28

**Authors:** Serap Keskin Tunç, Mehmet Emin Toprak, Esra Yüce, Nihat Efe, Celalettin Topbaş

**Affiliations:** 1 Department of Oral and Maxillofacial Surgery, Hamidiye Faculty of Dentistry, University of Health Sciences, İstanbul, 34668 Türkiye; 2 Department of Oral and Maxillofacial Surgery, Faculty of Dentistry, Gazi University, Ankara, 06510 Türkiye; 3 Department of Oral and Maxillofacial Surgery, Faculty of Dentistry, İstanbul Aydın University, İstanbul, 34295 Türkiye; 4 Department of Oral and Maxillofacial Surgery, Faculty of Dentistry, Van Yüzüncü Yıl University, Van, 65080 Türkiye; 5 Department of Endodontics, Hamidiye Faculty of Dentistry, University of Health Sciences , İstanbul, 34668 Türkiye

**Keywords:** Oral cancer, Dental education, Awareness, Knowledge, Early diagnosis

## Abstract

**Objective:**

This cross-sectional online questionnaire-based study evaluated the knowledge, awareness, and behaviors of dentists and senior dental students regarding oral cancer (OC).

**Materials and methods:**

This study included 168 dentists and senior dental students who had completed all theoretical educations and clinical practices. An online survey was administered to all participants to assess their awareness of the risk factors for OC, clinical knowledge, and behaviors. The participants’ demographic characteristics and knowledge of OC were analyzed.

**Results:**

Of the participants, 48.8% were female and 51.2% were male. Their mean age was 27.04 ± 5.56 years (range: 21–51). In addition, 59.5% were dentists, and 40.5% were senior dental students. The dentists’ mean time since graduation was 6.38 ± 5.64 years (range: 1–27). Routine oral mucosa examination for OC was significantly more frequent among the dentists than the senior dental students (*p* < 0.05). Among all participants, 33% of dentists and 51.5% of senior dental students had poor knowledge of OC-related or possibly predisposing factors. Routine evaluation of OC-related and predisposing risk factors, including human papillomavirus infection, smoking, alcohol use, trauma-related non-healing oral lesions caused by long-term incompatible prostheses, and poor oral hygiene, was significantly more frequent among the dentists than the senior dental students (*p* < 0.05).

**Conclusion:**

Educating dental students about a more comprehensive oral examination and early diagnosis of OC would help overcome the current lack of adequate knowledge and attitudes in OC prevention and early detection. A core curriculum compatible with the global standards on OC should be provided to dental students.

**Supplementary Information:**

The online version contains supplementary material available at 10.1186/s12903-024-04241-6.

## Introduction

Oral cancer (OC) is a malignancy affecting the lips, palate, floor of the mouth, gingiva, alveolar mucosa, buccal mucosa, or oropharynx [1]. It is the sixth most common cancer worldwide and the ninth leading cause of cancer-related mortality [2]. Oral cavity cancers are the second most common type of head and neck malignancy after skin and thyroid cancer in Türkiye [3]. Among cancer-related deaths, OC ranks twentieth in men and sixteenth in women worldwide [4]. Almost half of patients with OC and non-survivors are aged ≥ 65 years [1]. 

OC has a multifactorial etiology; excessive alcohol consumption, heavy smoking, and betel nut chewing are among its main risk factors [5]. The risk of OC is 13-fold higher in individuals with simultaneous exposure to alcohol and smoking [6]. In addition, human papillomavirus (HPV)-16 and HPV-18 infections are the primary risk factors for oropharyngeal cancer, while exposure to ultraviolet radiation is the leading risk factor for lower lip cancer [7]. Other risk factors include immunodeficiency, malnutrition, and low socioeconomic status [8]. Despite recent advancements in diagnosing and treating OC, nearly half of patients die within the first five years after diagnosis; however, the survival rate can increase to 70–90% with early diagnosis and effective treatment [9]. 

In its early stages, OC may be asymptomatic or resemble benign lesions. It is diagnosed mainly based on the clinician’s examination findings and a lesion biopsy [10]. Early diagnosis is theoretically possible since the mouth is a visible part of the human body. However, insufficient knowledge and low awareness of OC may result in late diagnosis. About half of OCs are diagnosed late, highlighting the importance of awareness and knowledge on this issue [11]. 

Several studies have investigated the behaviors and knowledge of dentists and dental students about OC [7, 12–14]. All these studies have underscored the need for dentists to improve the early diagnosis and prevention of OC. Most studies worldwide have conducted questionnaire surveys to determine dentists’ awareness and knowledge [4, 15]. However, only a few studies have examined this topic in Türkiye. Therefore, this study aimed to assess the awareness, knowledge, and behaviors regarding OC among dentists working at universities, state oral and dental health centers, and private clinics and senior dental students.

## Materials and methods

### Study design

The study group included senior dentistry students in the Faculty of Dentistry at Van Yüzüncü Yıl University (VYYUFD) who had completed all theoretical and clinical practice educations and dentists in Türkiye. This cross-sectional online-based questionnaire survey study was conducted between June and July 2020 and reported according to the Checklist for Reporting Results of Internet E-Surveys (CHERRIES) guidelines [16, 17]. The survey targeted senior dental students at the VYYUFD and dentists whose email addresses were eligible. In total, 180 dentists and senior dental students were deemed eligible participants. All participants were informed about the study’s aim and provided informed consent.

After reviewing previous studies [18–20], an online questionnaire was developed and used in this study. The survey was pilot-tested with 30 undergraduate students and dentists to verify its simplicity and clarity. Two experts in oral medicine (SKT, MET) evaluated the validity of the questionnaire content, which was pre-validated in previous research studies [18, 19]. 

The survey’s reliability was assessed using the testing and retesting method, in which 30 senior dental students and dentists completed the questionnaire twice within two weeks. Both outcomes were compared using Pearson’s correlation coefficient, which showed a significant stability coefficient, suggesting good test-retest reliability. The internal consistency between items in the survey was evaluated using Cronbach’s alpha. A Cronbach’s alpha of 0.80 was attained, indicating acceptable internal consistency.

An online questionnaire was created using Google Drive, and a link was emailed and shared with all eligible participants in closed groups. A cover letter accompanying the questionnaire explained the study’s aims and methods and assured that participation was anonymous and voluntary and that all provided information would be kept confidential and used for research purposes only. The questionnaire consisted of close-ended questions that collected information on the demographics, knowledge, practices, and attitudes toward OC prevention and early detection among senior dental students and dentists.

The study protocol was approved by the XXX University Non-Invasive Clinical Research Ethics Committee (approval no.: XXX). This study was conducted according to the principles in the Declaration of Helsinki.

### Data collection

The survey questions collected information on participants’ demographics (e.g., age, sex, and education level) and knowledge and attitudes toward OC prevention and early detection. The knowledge questions asked participants about their knowledge regarding OC symptoms, signs, and risk factors (16 questions). Each question answered correctly was given a score of “1.” The overall knowledge score ranged from 0 to 16, and this study used (9.6 / 16) 60% as the cut-off; [18, 21] a score > 9.6 points was considered good knowledge of OC, and a score *≤* 9.6 points was considered poor knowledge.

The third part of the questionnaire included 18 items asking participants about identifying potential clinical manifestations of OC. Each potential manifestation for regular OC screening had a “Yes” or “No” checkbox. The answers of dentists and senior dental students for each question were recorded and compared between groups.

In the final part of the questionnaire, the participants were asked about their attitudes and opinions toward OC. The questions evaluated the participants’ specialist choice for consultation of highly suspicious lesions, self-evaluation of good knowledge and adequate education on the early diagnosis and prevention of OC, intention or demand for further education and training on OC, and likely choice for the type of education/training.

### Statistical analysis

Statistical analyses were performed using the Number Cruncher Statistical Systems software (version 2007; NCSS, LLC, Kaysville, UT, USA). Descriptive data were expressed in the mean ± standard deviation (SD), median (min–max), or number (percentage), as applicable. The normality of the data distribution was assessed using the Shapiro–Wilk test and box plots. The non-normally distributed variables were compared between groups using the Mann–Whitney U test. The qualitative variables were compared between groups using the chi-square, Fisher’s exact, and Fisher–Freeman–Halton tests. A *p*-value of < 0.05 was considered statistically significant.

## Results

The response rate to the online survey was 93.3%, with 100 dentists (59.5%) and 68 (40.5%) senior dental students completely answering all items; 12 participants who answered the survey incompletely were excluded. Of the 168 participants, 48.8% were female and 51.2% were male. Their mean age was 27.04 ± 5.56 (range: 21–51) years. The dentists’ mean time since graduation was 6.38 ± 5.64 (range: 1–27) years. The participants’ descriptive data are summarized in Table 1.

**Table 1:** Descriptive data of the participants

Age (year)	*min-max (median)*	21–51 (25)
	*mean ± SD*	27,04 ± 5,56
**Sex**	**Female**	82 (48,8%)
	**Male**	86 (51,2%)
**Graduation status**	**Graduate**	100 (59,5%)
	**Senior student**	68 (40,5%)
**Time from graduation for dentists (year) (** ***n*** ** = 100)**	*min-max (median)*	1–27 (5)
	*mean ± SD*	6,38 ± 5,64

SD: standard deviation; ODHC: oral and dental health centers

Among participants, 69% performed a routine oral mucosa examination. Of the 31% who did not perform a routine oral mucosa examination, 92.3% conducted oral mucosa screening if they thought the patient was at high risk for potential oral malignancy. The participants’ behaviors and knowledge regarding OC-related or predisposing factors according to graduation status were examined (Table 2). Among participants, 53% provided information about OC-related or predisposing risk factors to their patients, and 63.7% performed a further examination for those with a suspicious oral lesion. In addition, 33% of the dentists and 51.5% of the dental students had poor knowledge of OC-related or possibly predisposing factors.

**Table 2:** The comparison of behaviors and knowledge level of the dentists and senior dental students on possible oral cancer-related or predisposing factors

	Graduate (*n* = 100)		Undergraduate(*n* = 68)	^a^ *p*
Routine oral mucosa examination				
**Yes**	75 (75%)	41 (60,3%)		0,043*
**No**	25 (25%)	27 (39,7%)		
**• Possible oral cancer-related or predisposing risk factors**				
** Smoking**	78(78%)	41(60,3%)		0,013*
** Alcoholism**	37(37%)	11(16,2%)		0,003**
** Sun exposure**	7(7%)	2(2,9%)		^b^0,145
** HPV, AIDS, immunocompromised patients**	4(4%)	3(4,4%)		0,066
** Long-term incompatible prostheses’ trauma related non-healing oral lesions**	40(40%)	5(7,4%)		0,001**
** Leukoplakia, white and erythematous lesions**	4(4%)	3(4,4%)		^b^1,000
** Malignancy**	0(0%)	3(4,4%)		^b^0,065
** Genetic predisposition**	18(18%)	12(17,6%)		0,953
** Gingival and mucosal pigmentation with irregular borders**	0(0%)	1(1,5%)		^b^0,405
** Systemic diseases**	10(10%)	9(13,2%)		0,801
** Chemotherapy, radiation therapy**	6(6%)	4(5,9%)		^b^1,000
** Stress**	5(5%)	4(5,9%)		^b^1,000
** Poor oral hygiene**	21(21%)	5(7,4%)		0,007**
** Malnutrition**	5(5%)	0(0%)		^b^0,082
** Age or sex predisposition**	3(3%)	2(2,9%)		^b^0,648
** Unknown**	5(5%)	11(16,2%)		0,028*
**Informing patients about oral cancer-related or predisposing risk factors**				
** Yes**	53 (53%)	36 (52,9%)		^b^1,000
** No**	47 (47%)	32 (47,1%)		
**Further examination for suspicious lesions**				
** Yes**	67 (67%)	40 (58,8%)		0,279
** No**	33 (33%)	28 (41,2%)		
**Level of knowledge on oral cancer-related or predisposing factors**				
** Poor knowledge**	33 (33%)	35 (51,5%)		
** Good knowledge**	67 (67%)	33 (33,8%)		

*n* = number ^a^Pearson chi-square test ^b^Fisher’s exact test ^c^Fisher-Freman-Halton test ^d^Mann-Whitney U test. •A multiple-choice item. Data are given in mean ± SD, median (min-max) or number and frequency, unless otherwise stated. HPV: human papilloma virus; AIDS: acquired immunodeficiency syndrome. *p < 0,05 **p < 0,01

Significantly more dentists than senior dental students performed routine oral mucosa examinations for OC (*p* < 0.05). Additionally, significantly more dentists than senior dental students performed routine evaluations of OC-related and predisposing risk factors, including HPV, smoking, alcohol use, trauma-related non-healing oral lesions caused by long-term incompatible prostheses, and poor oral hygiene (*p* < 0.05). However, other risk factors did not differ significantly between the dentists and senior dental students (*p* > 0.05).

The dentists were significantly better able to identify potential clinical manifestations of OC based on the pattern, size, and changes of the lesion, non-healing wounds, treatment-refractory lesions, painful/painless ulcerative lesions, diffuse erythematous lesions, and irregular lesions with obscure margins than the senior dental students (*p* < 0.05). However, the ability to identify other items did not differ significantly between the dentists and senior dental students (*p* > 0.05). The participants’ awareness of potential clinical manifestations of OC was also evaluated using an 18-item scale. It was found to be significantly higher among the dentists than the dental students (*p* < 0.01; Table 3).

**Table 3:** Comparison the identification of potential clinical manifestations of oral cancer by the participants

	Graduate (*n* = 100)	Undergraduate(*n* = 68)	^a^p
•Clinical characteristics of oral cancers			
**Mucosal irregular discoloration**	37(37%)	27(39,7%)	0,723
**Pattern, size, and shape changes of lesion**	9(9%)	1(1,5%)	^***b***^ 0,050*
**Oral cavity tissue growth and hyperplasia**	19(19%)	13(19,1%)	^b^1,000
**Spontaneous bleeding**	7(7%)	8(11,8%)	0,288
**Non-healing and treatment-refractory lesions**	26(26%)	4(5,9%)	0,001**
**Swelling, erythema**	6(6%)	8(11,8%)	^b^0,185
**White lesions**	10(10%)	10(14,7%)	^b^0,355
**Precancerous lesions, leukoplakia, erythroplakia**	9(9%)	4(5,9%)	^b^0,458
**Painful/painless ulcerative lesions, diffuse erythematous lesions**	23(23%)	4(5,9%)	0,003**
**Irregular lesions with obscure margins**	9(9%)	1(1,5%)	^***b***^ 0,050*
**Spontaneous painful/painless lesions**	9(9%)	3(4,4%)	^b^0,364
**Aphthous ulcers/lesions, pigmentations**	6(6%)	3(4,4%)	0,740
**Numbness of the area of the mouth**	2(2%)	3(4,4%)	^b^0,395
**Irregular lesions of the tongue or floor of the mouth leading to swallowing or chewing difficulties**	6(6%)	2(2,9%)	^b^0,476
**Lesions disrupting the mucosal integrity**	5(5%)	1(1,5%)	^b^0,403
**Solid areas with keratinization or necrosis**	6(6%)	2(2,9%)	^b^0,476
**Jaw bone expansion, tooth displacement/mobility without an apparent cause, irregular bone loss**	4(4%)	2(2,9%)	^b^1,000
**Idiopathic**	15(15%)	10(14,7%)	0,958
**Potential clinical manifestations of oral cancer awareness score**			
** min-max (median)**	0–6 (2)	1–4 (1)	^***d***^ 0,002**
** mean ± SD**	2,08 ± 1,13	1,55 ± 0,78	

n = number ^a^Pearson chi-square test ^b^Fisher’s exact test ^d^Mann-Whitney U test. •A multiple-choice item. Data are given in mean ± SD, median (min-max) or number and frequency, unless otherwise stated. ^*^p < 0,05 ^**^p < 0,01

The participants’ attitudes toward patients with suspected OC are shown in Table 4. Among them, 74.4% reported that a dentist should examine oral lesions, and 25.6% preferred a physician. The participants’ choice of specialist for consultation for a patient with a highly suspicious OC were primarily oral and maxillofacial surgeons (43.4%), another dentist (26.2%), or ear, nose, and throat specialists (20.6%). In addition, 16.1% (*n* = 27) had sufficient knowledge regarding the early diagnosis and prevention of OC. Among participants, 98.8% (*n* = 166) needed further education and information about OC, with 13.3% (*n* = 22) preferring an information package, 23.5% (*n* = 39) preferring training courses, and 63.3% (*n* = 105) preferring seminars on this topic.

**Table 4:** The attitudes of the participants for patients with suspected oral cancer

Professionals for oral lesions	Dentist	125(%74,4)
	**Physician**	43(%25,6)
**•Specialist choice for consultation of highly suspicious lesion for OC**	**Another dentist**	84(%26,2)
	**Ear, nose and throat specialist**	67(%20,6)
	**Oral and maxillofacial surgeon**	141(%43,4)
	**General practitioner**	4 (%1,2)
	**Plastic surgeon**	14 (%4,3)
	**Other**	14 (%4,3)
**Self-evaluation on good knowledge and adequately education on early diagnosis and prevention of oral cancers**	**Yes**	27 (%16,1)
	**No**	141(%83,9)
**Intention or demand for further education and training on oral cancers**	**Yes**	166(%98,8)
	**No**	2(%1,2)
**Probably choosing the type of education/training (** ***n*** ** = 166)**	**Information package**	22(%13,3)
	**Training courses**	39(%23,5)
	**Seminars**	105(%63,3)

•A multiple-choice item. Data are given in number and frequency, unless otherwise stated

## Discussion

Despite recent developments in the diagnostic and therapeutic modalities for managing and surgically treating OC, five-year overall survival rates have remained below 50% for many decades [2]. Routine oral examinations by dentists and consultations for patients with suspected lesions are helpful for early diagnosis of OC. Dentists’ awareness of OC risk factors and lifestyle modifications can help to prevent OC and improve survival rates among patients [18]. Many studies worldwide have investigated dentists’ knowledge, awareness, and practices for preventing and early diagnosing OC [13, 15–41]; however, no study has compared the knowledge levels of dentists and senior dental students regarding OC.

The survey response rate among participants with delivery confirmation appears higher in our study (93.3%) than in previous studies using online surveys [13, 25, 42]. We believe this difference is due to the following reasons. In this cross-sectional study, we administered a questionnaire to our own dental students (SKT and MB). We also administered the questionnaire to dentists who resided in the same region. This high participation rate was achieved with the support of the city’s dental association chamber. This difference can also be attributed to the greater tendency of the specifically selected study population to participate in scientific research and the use of a reliable data collection method. We consider these to be the reasons for the higher response rate in our study compared to previous studies [13, 21, 24–29, 34, 42]. 

The rate of early OC diagnosis is higher among dentists than physicians since they are provided detailed education on oral cavity examination. Macpherson et al. [43] reported that 58% of dentists performed a routine OC examination as part of the dental examination, and 48% could assess whether a suspected lesion required urgent referral. Hollows et al. [44] reported that only 40% of patients with suspicious oral lesions referred to a physician by their dentist sought advanced medical treatment. Physicians were the most contacted healthcare professionals for patient groups with limited access to healthcare services, including older adults, those with alcoholism and/or tobacco abuse, and those with low socioeconomic status.

The core curriculum of dentistry faculties in Türkiye has lacked standardization for many years, and various curricula have been adopted. However, in 2016, the Council of Higher Education in the Republic of Türkiye issued the Undergraduate Dental Education National Core Curriculum. This framework outlines the basic components and principles of dental education to facilitate competence- and qualification-based learning and the adoption of a well-designed education program that meets the minimum competencies in compliance with international standards [45]. The scope of this core curriculum was to provide education regarding malignant neoplasms of the oral cavity, their associated risk factors, signs, and symptoms and to improve the skills of dental students for suspecting malignancies and referring patients with preliminary diagnoses to a specialist.

A literature review highlighted the unmet need for special education programs and experience for OC screening, including among primary care healthcare professionals. Several studies have shown that most healthcare professionals are willing to be educated on examination techniques, signs and symptoms (non-healing ulcers and red or white spots of unknown origin), preventive measures (lifestyle modifications, smoking cessation, and limiting alcohol consumption), and referral issues [43, 46, 47]. In our study, only 3% of participants were very well informed about the clinical appearance of OC during their education. This finding underscores the importance of including up-to-date data on risk factors in the curricula of dentistry faculties and improving continuing education for dental professionals after graduation.

The role of some risk factors in OC development has been proven, and many studies have investigated the level of awareness of primary care physicians and dentists of these risk factors and their diagnostic value, reporting similar results [43, 48]. Greenwood and Lowry [27] found that dentists identified tobacco usage (93.7%) and alcohol consumption (85.3%) as the main risk factors for OC, respectively. In our study, despite their differing education levels, most participants identified the potential risk factors as tobacco/alcohol usage, previous OC, and sun exposure. However, Pavão Spaulonci et al. reported that 90–100% of Brazilian dentists could identify the potential risk factors as tobacco/alcohol use, HPV, and sun exposure [42]. Other large-scale surveys by Amer et al. [49] and Formosa et al. [50] showed similar results for identifying risk factors for OC. Unlike these studies, in our study, tobacco use, alcohol consumption, HPV, and sun exposure were identified as the main risk factors for OC. Notably, these rates were relatively higher than the results of a Malaysian study by Bhagavathula et al. [51] in which only 61% of the dentists and 14% of the dental students identified tobacco and alcohol as OC risk factors. These findings suggest that the level of awareness about the hazardous effects of tobacco and alcohol use and sun exposure among dentists remains low in Türkiye. Therefore, dental professionals should be provided with more comprehensive education or continuing education on OC-related risk factors.

HPV has been identified as a major risk factor for oropharyngeal squamous cell carcinoma (SCC) [52]. HPV-related SCCs have a unique pathophysiology; as such, SCC caused by tobacco use can be treated more effectively with combined chemotherapy and radiation therapy [53]. In our study, very few participants were able to identify HPV as a risk factor for OC, emphasizing the need to increase awareness of HPV and its preventive measures, such as vaccination, among dental professionals through the curricula of the dentistry faculties.

In our study, 7.4% of the senior dental students and 40% of the dentists identified non-healing lesions and chronic trauma caused by incompatible prostheses as risk factors for OC. While the difference was significant between the dentists and dental students, the rate of participants who could identify trauma due to incompatible prostheses as a risk factor for OC was relatively low. Some authors have advocated that injuries caused by incompatible prostheses do not cause OC; however, such injuries may mask the true symptoms of malignancy by changing the appearance of the oral cavity and delaying diagnosis [42, 49]. In our study, 4% of the senior dental students and 21% of the dentists identified poor oral hygiene as a potential risk factor for OC. This finding is consistent with Oji and Chukwuneke [54], who reported that poor oral hygiene was strongly associated with OC. However, further large-scale, prospective studies are warranted to elucidate its role in OC development.

In our study, 21% of dentists also identified malnutrition as a risk factor for OC. However, none of the dental students identified malnutrition as a risk factor, indicating a significant difference between the groups. Debate regarding the possible role of malnutrition in the development of all cancers, including OC, is ongoing. Shivappa et al. [55] investigated the inflammatory potential of diet and risk of oropharyngeal cancer in the Italian population, finding that a proinflammatory diet and tobacco/alcohol use correlated positively with OC. However, Dholam and Chouksey [56] found no significant correlation between diet and OC. Another study by Scully [57] concluded that further randomized clinical trials were needed to elucidate the effectiveness of dietary supplementation in minimizing OC risk and eliminating the need for chemotherapy.

In our study, very few dentists and dental students identified stress as a risk factor for OC. While a recent study showed that emotional stress increased OC risk [42], Dholam and Chouksey [56] recognized that emotional stress was a symptom of modern life and individuals refrained from seeking immediate help due to commitments at work or home, which might delay the diagnosis but not impact the etiology of OC. Similarly, our results indicate that emotional stress and malnutrition were considered secondary risk factors for OC in addition to well-established risk factors such as HPV, tobacco and alcohol use.

Our study also examined the identification of clinical manifestations of suspected lesions as the next step in diagnosing OC. The level of knowledge on the pattern, size, and changes of lesions, non-healing wounds, treatment-refractory lesions, painful/painless ulcerative lesions, diffuse erythematous lesions, and irregular lesions with obscure margins was significantly higher among the actively working dentists than among the dental students. In their study including only dental specialists, Kebapcioglu and Pekiner [7] reported that 35.9% considered small, painless, white areas as the clinical properties of OC, while 26.5% considered small, painless, red areas as the main signs of an OC lesion.

Vijay and Suresan [21] showed that most dentists (82%) considered non-scrapable, white lesions the most common manifestation, while only 9.6% considered red erosions the most common manifestation. Clovis et al. [30] reported that most dentists (77%) correctly identified small, painless, red lesions as precancerous OC lesions. In our study, the relatively low response rate could be attributed to inadequate knowledge of both the risk factors and clinical characteristics of OC.

An effective development plan should be created on early OC diagnosis and potentially malignant entities encountered in clinical practice and provided to both undergraduates and qualified professionals through continuing education [18]. Studies have shown that the rate of early OC diagnosis is significantly higher among dentists who attend training courses or conferences [34, 58]. Considering the importance and relevance of continuing education, 98.8% of our participants needed further education and information about OC. We believe that these findings contribute to the body of knowledge in the literature and pave the way for future studies examining the curricula of dentistry faculties.

Our study had some limitations related to its scope, potential biases, and the nature of its cross-sectional design. It primarily used close-ended questions, which may limit the depth of understanding regarding the reasons behind certain behaviors or attitudes and cause a lack of qualitative insights. It also relied solely upon an online survey, and participants who were not comfortable or proficient with the internet and digital tools could not be accessed. Its number of participants was also low, and larger study groups are needed for more robust results.

## Conclusion

In conclusion, the findings of this survey emphasize an unmet need for basic education about OC prevention and early detection at VYYUFD. Dentists had higher knowledge and behavior scores for OC prevention and early detection than senior dental students. Dental students at VYYUFD require more comprehensive education about OC to fill the gap and overcome current shortcomings. We believe all dental students should be provided with a clinical core curriculum on OC that is compatible with global standards.

### Electronic supplementary material

Below is the link to the electronic supplementary material.

Supplementary Material 1

Supplementary Material 2

## Data Availability

The datasets generated and/or analyzed during the current study have been shared. All data generated or analysed during this study are included in this published article [and its supplementary information files].
