# Supplementary material for: Comparison of knowledge, awareness, and behaviors toward oral cancer among dental students and dentists: an online cross-sectional questionnaire in Türkiye
Source: BMC Oral Health. 2024 Apr 28;24:502. doi: 10.1186/s12903-024-04241-6 (PMC11057109; doi:10.1186/s12903-024-04241-6)
Supplement: Supplementary file 2 — Supplementary Material 2 [file 12903_2024_4241_MOESM2_ESM.docx]

**Comparison of Knowledge, Awareness and Behaviors Toward Oral Cancer Among Turkish Dentists and Senior Dental Students**

This survey is prepared for to compare the knowledge, awareness and behaviors toward oral cancer among Turkish dentists and senior dental students. The senior dentistry students should be completed all theoretical and clinical practice educations at dentistry faculties in Türkiye. The participation will be volunteered and anonymously. A written informed consent will be obtained from each participant.

* Multiple choice is possible

- **Age**
- **Sex?**

O Male / O Female

- **Graduation status?**

O Senior dental student / O Dentist

- **Time from graduation date for dentists?**
- **Working place?**

O None (senior students)

ODHC

O Private clinic

O University

- **Specialty in dentistry?**

O Yes / O No

- **Field of specialty?**

O Oral dental and maxillofacial radiology

O Maxillofacial surgery

O Endodontics

O Orthodontics

O Pedodontics

O Periodontics

O Prosthetic dentistry

O Restorative dentistry

- **Routine oral mucosa examination?**

O Yes / O No

- **If you don’t perform routine oral mucosa examination do you perform oral mucosa screening in high-risk patients?**

O Yes / O No

- **Choose the oral cancer-related or predisposing risk factors? ***

O Smoking

O Alcoholism

O Sun exposure

O HPV, AIDS, immunocompromised patients

O Long-term incompatible prostheses’ trauma related non-healing oral lesions

O Leukoplakia, white and erythematous lesions

O Malignancy

O Genetic predisposition

O Gingival and mucosal pigmentation with irregular borders

O Systemic diseases

O Chemotherapy, radiation therapy

O Stress

O Poor oral hygiene

O Malnutrition

O Age or sex predisposition

O Unknown

- **Do you inform the patients about oral cancer-related or predisposing risk factors?**

O Yes / O No

- **What is the level of your knowledge on oral cancer-related or predisposing factors?**

O Poorly informed

O Adequately informed

O Well informed

O Very well informed

- **Do you identify the potential clinical manifestations of oral cancer?***

O Mucosal irregular discoloration

O Pattern, size, and shape changes of lesion

O Oral cavity tissue growth and hyperplasia

O Spontaneous bleeding

O Non-healing and treatment-refractory lesions

O Swelling, erythema

O White lesions

O Precancerous lesions, leukoplakia, erythroplakia

O Painful/painless ulcerative lesions, diffuse erythematous lesions

O Irregular lesions with obscure margins

O Spontaneous painful/painless lesions

O Aphthous ulcers/lesions, pigmentations

O Numbness of the area of the mouth

O Irregular lesions of the tongue or floor of the mouth leading to swallowing or chewing difficulties

O Lesions disrupting the mucosal integrity

O Solid areas with keratinization or necrosis

O Jaw bone expansion, tooth displacement/mobility without an apparent cause, irregular bone loss

O Idiopathic

- **The attitudes of the senior dental students / dentists for patients with suspected oral cancer**
- **Professional choice for oral lesions?**

O Dentist

O Physician

- **Specialist choice for consultation of highly suspicious lesion (malignancy)?**

O Another dentist

O Ear, nose and throat specialist

O Oral and maxillofacial surgeon

O General practitioner

O Plastic surgeon

O Other

- **Do you think that you adequately informed and your education on early diagnosis and prevention of oral cancers is sufficient?**

O Yes / O No

- **Your intention or demand for further education and training on oral cancers?**

O Yes / O No

- **What kind of education/training will be your choice?***

O Information package

O Training courses

O Seminars
